# An adaptive dimension differential evolution algorithm based on ranking scheme for global optimization

**DOI:** 10.7717/peerj-cs.1007

**Published:** 2022-06-17

**Authors:** Tien-Wen Sung, Baohua Zhao, Xin Zhang

**Affiliations:** Fujian Provincial Key Laboratory of Big Data Mining and Applications, College of Computer Science and Mathematics, Fujian University of Technology, Fuzhou, Fujian, China

**Keywords:** Global optimization, Differential evolution, Swarm intelligence, Adaptive dimension, Ranking scheme

## Abstract

In recent years, evolutionary algorithms based on swarm intelligence have drawn much attention from researchers. This kind of artificial intelligent algorithms can be utilized for various applications, including the ones of big data information processing in nowadays modern world with heterogeneous sensor and IoT systems. Differential evolution (DE) algorithm is one of the important algorithms in the field of optimization because of its powerful and simple characteristics. The DE has excellent development performance and can approach global optimal solution quickly. At the same time, it is also easy to get into local optimal, so it could converge prematurely. In the view of these shortcomings, this article focuses on the improvement of the algorithm of DE and proposes an adaptive dimension differential evolution (ADDE) algorithm that can adapt to dimension updating properly and balance the search and the development better. In addition, this article uses the elitism to improve the location update strategy to improve the efficiency and accuracy of the search. In order to verify the performance of the new ADDE, this study carried out experiments with other famous algorithms on the CEC2014 test suite. The comparison results show that the ADDE is more competitive.

## Introduction

Optimization exists in various fields of engineering and applications (Song, Pan & Chu, 2020; Wang, Pan & Chu, 2020), and the intelligent algorithm is one of the important ways to solve optimization problems (Zhang & Liu, 2019; Pan et al., 2021). In recent years, with the rapid development of artificial intelligence, more and more scholars begin to explore and study the optimization algorithms based on swarm intelligence. This kind of algorithms are useful and effective for the optimization problems in heterogeneous sensor networks (Wang et al., 2019; Elhoseny et al., 2015; Binh et al., 2020; Bhushan, Pal & Antoshchuk, 2018). The algorithms are also popular to be utilized in the sensor data and information processing (Moldovan et al., 2020; Awgner et al., 2020; Sheoran, Mittal & Gelbukh, 2020). For swarm intelligence-based algorithms, the differential evolution (DE) algorithm (Storn & Price, 1997), artificial bee colony (ABC) algorithm (Karaboga et al., 2007), and QUasi-Affine TRansformation Evolutionary (QUATRE) algorithm (Meng, Pan & Xu, 2016) algorithm have the characteristics of simple structure and ease of implementation. They are widely used in engineering practices and have become an important part of the contemporary optimization field. Among them, the DE algorithm attracts more attention because of its strong optimization ability and robustness.

To solve the problem of numerical optimization, Price and Storn firstly proposed differential evolution algorithm (DE) in 1995. Since the 21st century, more and more advanced DE variants have been proposed to be applied in different fields (Tanabe & Fukunaga, 2013a; Meng, Pan & Zheng, 2018; Meng & Pan, 2019; Meng, Pan & Tseng, 2019; Ali et al., 2016; Meng, Pan & Kong, 2018). It has become an effective technology to solve complex optimization problems. DE algorithm combines the idea of swarm intelligence into evolutionary computation and searches for the global optimal solution through the competition and cooperation of particles. In DE, the global optimization strategy based on population is adopted, and the differential operation is used for mutation operation. Through one-to-one competition, a better solution is generated, which improves the search efficiency of evolutionary computation. Also, the memory ability of DE can understand the current optimization situation and adjust the search strategy, so it has strong optimization ability and robustness, which is very suitable for solving some difficult optimization problems. In recent years, some new variants of DE (Price, Storn & Lampinen, 2006; Das & Suganthan, 2011; Das, Mullick & Suganthan, 2016) have also won the highest ranking in IEEE Evolutionary Computing Conference. However, DE has its shortcomings. For example, the crossover method determined by the parameter Crossover operator (CR) will lead to search bias. For this defect, Meng, Pan & Xu (2016) proposed the QUATRE algorithm (which can be regarded as an improved version of DE) that uses matrix M instead of CR to complete the crossover. Since the location update equation of the DE algorithm provides it with a strong exploit ability, the multi-dimensional update mode will make the DE algorithm easier to converge and fall into the local optimal solution. We know that multi-dimensional strategy will bring more exploit, especially in the early stage of the optimization process; it can approach the global optimal solution faster, while the one-dimensional update strategy has a slower search speed, but it can avoid falling into local optimum in the early stage of the search process. The artificial bee colony algorithm (ABC) is a kind of algorithm that adopts a one-dimensional update strategy and has strong search performance. The balance between exploration and exploit in optimization algorithm has always been a very difficult problem, and adaptive scheme (Islam et al., 2012; Ajithapriyadarsini, Mary & Iruthayarajan, 2019) is regarded as an effective technology to solve this problem. At present, the mainstream adaptive scheme of DE is realized by adjusting control parameters (Draa, Chettah & Talbi, 2019). In addition, there are also the adaptive dimension framework (Deng, Li & Sun, 2020) and the adaptive mutation strategy (Qin, Huang & Suganthan, 2009). These methods optimize the algorithm at different levels. They also have different adaptive trigger conditions, such as ranking schemes or reaching a certain number of times that cannot be updated. Among them, the sorting behavior is adopted by more and more scholars because it can intuitively reflect the advantages and disadvantages of the current individual and better integrate into the adaptive scheme. Among the research on ordering schemes, there are few adaptive strategies on dimension. Inspired by the single-dimensional updating strategy of the artificial bee colony algorithm, this study proposed a novel DE algorithm based on the ranking behavior (Cheng et al., 2021; Stanovov, Akhmedova & Semenkin, 2020; Tong et al., 2021; Xu et al., 2019; Leon & Xiong, 2018; Zhong, Duan & Cheng, 2021) and adaptive dimensional strategy (Deng, Li & Sun, 2020). The algorithm adaptively selects the update strategy according to the individual ranking, and combines the single dimension strategy with the multi-dimension strategy to achieve the balance of exploration and exploit.

The rest of this article is arranged as follows: in the second section, we briefly review the development of DE, the ABC algorithm, and their famous variants; The third section introduces the principle and structure of the proposed algorithm in detail. In “Experiment and Result”, experiments are carried out in the CEC2014 test suite (Liang, Qu & Suganthan, 2014), and the performance of the proposed algorithm is verified by comparing with the well-known variants of DE and ABC. Finally, the conclusion and prospects are given in “Conclusion”.

## Background review and related work

In this section, two important branches of the stochastic optimization algorithm, the famous ABC and the powerful DE, are reviewed as well as their variants.

### ABC algorithm and its variants

In the ABC algorithm, the global optimal solution is found through three different stages. In the first stage, the leader bee looks for a better honey source in the neighborhood of the honey source (feasible solution); in the second stage, the following bee selects one of the leading bees to follow and collect honey according to the information of the quality of the honey source found by the leader bee, so as to find a better honey source in the neighborhood of the honey source; in the third stage, when the leader bee cannot find a better honey source for many times, he will become a reconnaissance bee, The new honey source location was randomly searched within the search range. The standard functions of the location update process of the original ABC algorithm are shown as follows.

(1) $$\left\{ {\matrix{ {{\theta _{ij}} = {\theta _{j{\rm min}}} + r\cdot \left( {{\theta _{j{\rm max}}} - {\theta _{j{\rm min}}}} \right)} \cr {{x_{ij}}\left( {t + 1} \right) = {\theta _{ij}}\left( t \right) + \lambda \cdot \left( {{\theta _{ij}}\left( t \right) - {\theta _{kj}}\left( t \right)} \right)} \cr {{P_i} = F\left( {{\theta _i}} \right)/\mathop \sum \nolimits_{k = 1}^S F\left( {{\theta _k}} \right)} \cr } } \right.$$

where $i = 1,2, \ldots ,{\rm SN}$, $j = 1,2, \ldots ,D$, ${\theta _i}$ is the ith feasible solution with D dimension in SN, j is the dimension of every feasible solution, r is a random number and $r \in \left[ {0,1} \right]$. t is the number of iterations, ${\theta _k}$ is a randomly selected honeybee and $k \ne i$, *λ* is a random number and in the range of [−1,1], when the function value of the new honey source is better, the new honey source is used to replace the original honey source, otherwise the original honey source is retained. ${P_i}$ is the probability of selecting the ith hired bee, s is the number of hired bees, $\; {\theta _i}$ is the position of the ith hired bee, $F\left( {{\theta _i}} \right)$ is the fitness value of the ith bee, which is related to the objective function value of the corresponding honey source.

The original ABC algorithm updates the position by randomly approaching or away from other bees, which greatly enhances its search ability, but also weakens the exploitability and is not easy to converge to the global optimal solution. In the development of the ABC algorithm, many excellent ABC variants have been proposed (Bajer & Zorić, 2019; Cui et al., 2018; Peng, Deng & Wu, 2019; Wang et al., 2014; Li et al., 2020). For example, the global optimal guided GABC algorithm (Zhu & Kwong, 2010) adds the guidance of global optimal solution (Gbest) in the running formula, which effectively enhances the development ability of the algorithm. The authors who proposed this method improved the GABC algorithm again and proposed the IABC algorithm (Cao et al., 2019). The algorithm improves the search mechanism of the follower bee to solve the continuous optimization problem. Inspired by the differential evolution algorithm (DE), a modified ABC algorithm (MABC) (Gao & Liu, 2012) was proposed by combining the differential evolution algorithm with the ABC algorithm. The main idea of the MABC algorithm is to generate feasible solutions in the vicinity of the global optimal solution of the previous iteration in the process of position update. In the phase of population initialization and bee detection, chaos system and opposition based learning are used to enhance the development ability and global convergence performance of the algorithm. Inspired by the QUATRE algorithm, Zhao, Sung & Zhang (2021) proposed QABC algorithm in 2021. This algorithm uses multi-dimensional coevolution to complete the location update, which greatly enhances the development of the algorithm. However, because it replaces the original ABC update strategy, it also weakens the global search ability to a certain extent.

### DE algorithm and its variants

DE is a powerful evolutionary algorithm, the core idea of which is survival of the fittest. The evolution process of DE imitates the operation of mutation, crossover, and selection in genetics. It is a robust global optimization algorithm. The operation mechanism of DE consists of four steps (Storn & Price, 1997). First, starting from a randomly generated initial population, two individuals are selected from their parents to generate a difference vector. Second, another individual is selected to sum with the difference vector to generate the experimental individual. Third, the parent individual and the corresponding experimental individual are cross-operated to generate a new offspring individual. Finally, the selection operation is carried out between the parent generation individual and the offspring individual, and the individual that meets the requirements is saved to the next generation group. Through continuous evolution, good individuals are retained, poor ones are eliminated, and the search is guided to approach the optimal solution. Compared with crossover and selection, this variation has attracted more and more attention. As shown in Table 1, there are five common mutation strategies for the DE algorithm: DE/rand/1, DE/rand/2, DE/best/1, DE/best/2, and DE/target-to-best/1. They are used to generate new populations.

**Table 1:** Mutation strategies for the DE algorithm.

NO.	DE/x/y	Equation
1	DE/rand/1	$B = {X_{r1,G}} + F\cdot \left( {{X_{r2,G}} - {X_{r3,G}}} \right)$
2	DE/rand/2	$B = {X_{r1,G}} + {F_1}\cdot \left( {{X_{r2,G}} - {X_{r3,G}}} \right) + {F_2}\cdot \left( {{X_{r4,G}} - {X_{r5,G}}} \right)$
3	DE/best/1	$B = {X_{best,G}} + F\cdot \left( {{X_{r1,G}} - {X_{r2,G}}} \right)$
4	DE/best/2	$B = {X_{best,G}} + {F_1}\cdot \left( {{X_{r1,G}} - {X_{r2,G}}} \right) + {F_2}\cdot \left( {{X_{r3,G}} - {X_{r4,G}}} \right)$
5	DE/target-to-best/1	$B = {X_{i,G}} + {F_1}\cdot \left( {{X_{best,G}} - {X_{i,G}}} \right) + {F_2}\cdot \left( {{X_{r1,G}} - {X_{r2,G}}} \right)$

In the past 20 years, scholars have done a lot of research on DE, and many DE variants (Brest et al., 2006; Qin, Huang & Suganthan, 2009; Zhang & Sanderson, 2009; Tanabe & Fukunaga, 2013a, 2013b) have been proposed to enhance its performance. We know in the process of mutation and crossover that the choice of the parent will directly affect the quality of the offspring. Sutton proposed the RBDE algorithm (Sutton, Lunacek & Whitley, 2007), whose main improvement strategy is to optimize offspring individuals by changing the selection of parent individuals. In RBDE, the probability of being selected as a parent in the current population is different, and the better the individual is, the greater will be the probability of being selected. Some researchers do this by using a population structure. Das et al. (2009) proposed a DEGL algorithm using a new mutation strategy. Its main innovation point is that it adopts a neighborhood search mutation strategy based on the population loop topology structure. Moreover, its control parameters are generated randomly, and successful control parameters are more likely to be retained. Qin, Huang & Suganthan (2009) proposed a SADE algorithm based on adaptive strategy and crossover rate. Multiple strategies are gathered in SADE, in which the successful strategy is more likely to be selected and used. In each generation of crossover operation, its crossover rate is generated by the normal distribution of the average of the historical successful crossover rate. Meng, Pan & Xu (2016) proposed a swarm-based evolutionary algorithm called the QUATRE algorithm. Aiming at the inherent weakness of DE cross-selection in high-dimensional angle, this algorithm uses the collaborative search matrix M to replace the cross-control parameter CR in the DE algorithm. The individual then updates the position by the method of quasi-affine transformation, so as to solve the problem of position deviation in the search. Pan et al. (2017) proposed a new QUATRE algorithm based on the sorting method (S-QUATRE); its main innovation is that the population is divided into better and worse groups through a sorting strategy, the better group is directly reserved to the next generation, while the individuals in the worse group can only be passed to the next generation after evolution. Aiming at the problem that it is difficult for QUATRE to jump to the local optimal solution in the later stage of iteration, Sung, Zhao & Zhang (2021) proposed the QUATRE-DEG algorithm. The algorithm uses the dual population structure to maintain the diversity of information, and constructs a gravity model of global optimal solution and global suboptimal solution to coordinate the balance of exploration and exploit.

## Proposed algorithm

As we all know, search and development are equally important for the performance of swarm intelligence algorithms. DE is well known for its powerful development performance, which largely benefits from its multidimensional search strategy. In the search process, it can quickly approach the global optimal solution, but there will be premature convergence. This is caused by the lack of search performance in the late iteration, and the one-dimensional search strategy can make up for this deficiency. ABC algorithm is famous for its powerful searching ability. It uses a one-dimensional search strategy to update the location. Inspired by ABC, a new DE algorithm is proposed in this study, which update by the adaptive selection of single or multi-dimensional search strategy. The arrangement of this chapter is as follows: In the first part, the improved location update equation is given. In the second part, the adaptive selection scheme for the multi-dimensional update or single dimensional update is described. Finally, the pseudo-code of the proposed ADDE algorithm is given in the third section.

### Improved location update strategy

Generally speaking, elitism is an effective technique to enhance the performance of the algorithm in the optimization process: using elitist individuals (better individuals) to guide other individuals to complete the evolution, will more quickly approach the global optimal solution. However, when only one elite is used, for example, only the global optimal position of the current iteration is used for guidance, it is easy to fall into the local optimal solution and converge prematurely.

(2) $${x_{ij}}\left( {t + 1} \right) = {x_{ij}}\left( t \right) + \lambda \cdot \left( {{x_{ij}}\left( t \right) - {x_{kj}}\left( t \right)} \right) + \beta \cdot \left( {{x_{bj}}\left( t \right) - {x_{ij}}\left( t \right)} \right)$$

where ${x_i}$ is a random selection of other individuals different from the current individual, ${x_b}$ is a randomly selected individual that is better than the current individual, $\lambda$ is a random number generated between [−1,1], $\beta$ is a random number generated between [0,1.5]. As we can see, this update mechanism increases the guidance of better individuals. Better individuals are randomly generated, not only the optimal individuals, which undoubtedly brings more possibilities for search. The new mutation equation is as follows:

(3) $$B = {X_{b,G}} + F\cdot \left( {{X_{r2,G}} - {X_{r3,G}}} \right)$$

where ${X_b}$ is also a better individual randomly selected than the current individual, ${X_{r2}}$ and ${X_{r3}}$ are a random selection of individuals in the population and is different from each other. $F$ is the scaling factor used to control the influence of the difference vector, and $B$ is the generated experimental population. Similarly, ${X_{b,G}}$ can bring better experimental variables for DE, while still maintaining its randomness.

### Adaptive dimension selection scheme

In the second stage of the ABC algorithm, it is easier for observation bees to follow the better bees to collect honey. This means that the better the individual is, the easier it is to be selected for one-dimensional location updating. This is undoubtedly beneficial to the whole optimization process. The difference is that the poor individuals in the population usually mean that they are far away from the global optimal solution, and multidimensional updating can speed up the whole optimization process. Based on this, this article proposes an adaptive selection scheme based on ranking. Its selection mechanism is as follows:

(4) $${P_i} = ran{k_i}/NP$$

(5) $$\left\{ {\matrix{ {{x_{ij}}\left( {t + 1} \right) = {x_{ij}}\left( t \right) + \lambda \cdot \left( {{x_{ij}}\left( t \right) - {x_{kj}}\left( t \right)} \right) + \beta \cdot \left( {{x_{bj}}\left( t \right) - {x_{ij}}\left( t \right)} \right),\; if\; rand > {P_i}} \cr {B = {X_{b,G}} + F\cdot \left( {{X_{r2,G}} - {X_{r3,G}}} \right),\; if\; rand < {P_i}} \cr } } \right.$$

(6) $${u_{ij}} = \left\{ {\matrix{ {{B_{ij}},\; \; \; if\; rand \le CR\; or\; j = k} \cr {{x_{ij}}\left( t \right),\; \; \; \; \; \; \; \; \; \; \; \; \; \; \; \; \; \; \; else} \cr } } \right.$$

Among them, $ran{k_i}$ is the current individual ranking, The best individual $rank$ is one and the worst individual $rank$ is $NP$. $NP$ is the total number of particles in the population, and $rand$ is a random number between [0,1]. ${x_{ij}}\left( {t + 1} \right)$ is the progeny individual generated by the one-dimensional update strategy. $B$ is the experimental population generated by the multi-dimensional update strategy, it still needs to complete the crossover operation using Eq. (6) to generate the progeny individual ${u_{ij}}$ $CR \in \left( {0,1} \right)$ is the cross control parameter, and $k \in \left( {1,2, \ldots ,D} \right)$ is a random parameter index to ensure that each individual always selects at least one parameter from the experimental individuals.

As shown above, the higher the current individual ranking, the easier it is to choose a single-dimensional update strategy, while the lower the ranking, the easier it is to choose a multi-dimensional update strategy. In particular, when using a multi-dimensional strategy, there is still a need for crossover, and when using a single-dimensional strategy, there is no need for crossover. In the adaptive dimension selection scheme, according to the ranking of multi-dimensional or single dimensional update strategy selection, is a kind of effective play to their expertise technology, to achieve the balance of search and development.

### Algorithm of ADDE

**Algorithm:** ADDE

**INPUT:**	Bounds: [ $X_{min}^D$, $X_{max}^D$], Objective function: ***f****(*X*)*, Maximal generation number: $\; MaxGen$, Population size: $NP$
**OUTPUT:**	Individual of global optimal solution: ${x_{gbest}}$, global optimal value *f*( ${x_{gbest}}$).
1:	Initialize the population within the bounds, and calculate the fitness of all individuals.
2:	Set relevant parameters: ${\rm F}$, ${\rm CR}$.
3:	**for***g* = 1 to $MaxGen$
4:	All individuals were ranked according to fitness values.
5:	**for***i* = 1 to *NP*
6:	Randomly select the better individual than the current one.
7:	Calculating the probability of current individual adaptive selection ${P_i}$ using Eq. (4).
8:	**If** $rand > {P_i}$
9:	Generating test vectors using Eq. (2).
10:	**else**
11:	Generating experimental vectors using Eq. (3).
12:	Complete the crossover operation using Eq. (6).
13:	**end if**
14:	Keep better solutions based on greedy selection strategy.
15:	**end for**
16:	**end for**
17:	return: ${x_{gbest}}$ and f( ${x_{gbest}}$).

## Experiment and result

In our experiments, the CEC2014 benchmark set is used to evaluate the performance of the proposed algorithm. In the test benchmark suite, all benchmark functions can be divided into four categories: single-mode function ( ${f_1} - {f_3}$), basic multi-mode function ( ${f_4} - {f_{16}}$), mixed-function ( ${f_{17}} - {f_{22}}$), and combined function ( ${f_{23}} - {f_{30}}$). All basic functions are treated as black-box problems. According to Liang, Qu & Suganthan (2014), the search range is set to [−100,100].

It can be seen from the method part of Chapter 3 that the inspiration for the improvement of ADDE algorithm is the ABC algorithm. The inspiration mainly comes from two aspects. One is that the one-dimensional update equation of ABC brings greater search, and the other is that in the following bee stage of ABC algorithm, the individual position with higher (better) fitness value will get more position update opportunities. These two improvements are reflected in the location update strategy and adaptive selection scheme of the ADDE algorithm. Therefore, we compare our algorithm with some famous ABC variants and advanced DE variants to verify the effectiveness of our algorithm. ABC variants include: GABC, MABC, IABC, QABC, and DE variants include: DE/rand/1, RBDE, DEGL, SADE, QUATRE, S-QUATRE, QUATRE-DEG. The parameters of all algorithms are in line with the original recommended values, and the detailed parameters are listed in Table 2.

**Table 2:** Parameter settings for experiment.

Algorithm	Initial parameter settings
GABC	$NP = 50$, *limit* = $D*NP/2$
MABC	$NP = 50$, *limit* = $D*NP/2$
IABC	$NP = 50$, *limit* = $D*NP/2$, $MR$ = 0.3
QABC	$NP = 50$, *limit* = $D*NP/2$, $q$ = 0.6
DE/rand/1	$NP = 100,\; \; \alpha = \beta = F = 0.7$, $\; CR = 0.5$, ${\rm neighborhood\; size} = 0.1*NP$
RBDE	$NP = 100,\; \; F = 0.7$, $CR = 0.5$, $\beta = 3.0$
DEGL	$NP = 100,\; \; \alpha = \beta = F = 0.7$, $\; CR = 0.5$, ${\rm neighborhood\; size} = 0.1*NP$
SADE	$NP = 100$, $F{\sim}N\left( {{\mu _F},0.3} \right),\; \; {\mu _F} = 0.5,\; \; CR{\sim}N\left( {{\mu _{CR}},0.1} \right),\; \; {\mu _{CR}} = 0.5,\; \; LG = 50$
QUATRE	$NP = 100,\; F = 0.7$
S-QUATRE	$NP = 200,\; F = 0.7$
QUATRE-DEG	$NP = 100,\; \; F = 0.7$, $z = 0.4$
ADDE	$NP = 100,F = 0.5$, $CR = 0.9$

All experiments are carried out on the Windows 10 operating system with 8 GB memory and Intel i5-4210m processor. The simulation platform of the algorithm is MATLAB 2019a. For the reliability of the data, we conducted 51 independent experiments on all the comparison algorithms and collected the fitness error $\Delta f = {f^*} - f\left( O \right)$ of these test functions, where ${f^*}$ represents the final value of algorithm convergence, and $f\left( O \right)$ represents the global optimal value of the test function. In each run, the total number of function evaluations, *maxFES*, is set to 10e4*D, where *D* is the dimension. We then compared the average value of fitness error *∆f* and *std* (standard deviation) of 51 runs of each algorithm. In order to evaluate the significance of the two algorithms, a paired nonparametric statistical procedure of Wilcoxon’s signed rank test, was performed on experimental data (Derrac et al., 2011). The test method has been widely used by scholars to compare the results of two different populations (Deng, Li & Sun, 2020). In our experiment, the significance level was set at 0.05, and the comparison results, the symbols “+”, “−”, and “=” respectively represent that the performance of the new algorithm ADDE is “better”, “worse”, or “similar” than that of other algorithms.

### Comparison between ADDE and ABC variants

In this section, we compare with several well-known ABC variants, GABC, MABC, IABC, and QABC, and the statistical results based on Wilcoxon’s rank sum test are recorded in Tables 3–5. As we can see from Tables 3 to 5, firstly, in terms of the 10D real number optimization, the novel ADDE performs better than GABC on 21 benchmark functions and performs similar on two benchmark functions, better than IABC on 20 benchmark functions and performs similar on three benchmark functions, better than MABC on 23 benchmark functions and performs similarly on two benchmark functions, better than QABC in 22 benchmark functions and similar in four benchmark functions. Secondly, in terms of the 30D real number optimization, the novel ADDE performs better than GABC in 21 benchmark functions and similar in two benchmark functions, better than IABC in 23 benchmark functions and similar in two benchmark functions, better than MABC in 27 benchmark functions and similar in two benchmark functions, and better or similar in all 30 benchmark functions than QABC. Finally, in terms of the 50D real number optimization, the novel ADDE performs better than GABC in 21 benchmark functions and performs similar in one function, better than IABC in 25 benchmark functions and performs similar in one function, better than MABC in 26 benchmark functions and performs similar in one function, better than QABC in 23 benchmark functions and performs similarly in one function. In general, the performance of our algorithm is better than that of all the ABC variants.

**Table 3:** Comparison with ABC variants under *D* = 10.

	ADDE	GABC	IABC	MABC	QABC
*f*	Mean/std	Mean/std	Mean/std	Mean/std	Mean/std
${f_1}$	1.62E−13/2.0E−13	1.01E+05/6.8E+04(+)	3.14E+05/1.3E+05(+)	1.34E+05/6.7E+04(+)	**5.85E−15/8.6E−15(−)**
${f_2}$	**0.00E+00/0.0E+00**	3.50E+01/4.3E+01(+)	4.47E+02/5.1E+02(+)	1.08E+02/1.5E+02(+)	**0.00E+00/0.0E+00(=)**
${f_3}$	**0.00E+00/0.0E+00**	1.60E+02/1.3E+02(+)	1.44E+02/8.5E+01(+)	1.50E+02/2.5E+02(+)	**0.00E+00/0.0E+00(=)**
${f_4}$	1.53E+01/1.7E+01	**6.77E−02/1.3E−01(−)**	1.12E+00/1.7E+00(−)	1.16E+00/1.4E+00(−)	2.34E+01/1.6E+01(+)
${f_5}$	1.61E+01/6.7E+00	1.90E+01/4.3E+00(+)	**1.40E+01/8.8E+00(−)**	1.99E+01/1.2E+00(+)	1.82E+01/6.1E+00(+)
${f_6}$	**0.00E+00/0.0E+00**	1.20E+00/5.9E−01(+)	3.61E−01/2.9E−01(+)	6.54E−01/4.2E−01(+)	5.88E−01/7.8E−01(+)
${f_7}$	1.47E−02/2.1E−02	**6.35E−03/8.7E−03(−)**	1.36E−02/1.3E−02(−)	1.36E−02/1.5E−02(−)	8.29E−02/4.6E−02(+)
${f_8}$	**0.00E+00/0.0E+00**	**0.00E+00/0.0E+00(=)**	**0.00E+00/0.0E+00(=)**	**0.00E+00/0.0E+00(=)**	8.58E−01/8.4E−01(+)
${f_9}$	**3.63E+00/1.1E+00**	4.18E+00/1.4E+00(+)	5.25E+00/1.5E+00(+)	9.07E+00/2.0E+00(+)	5.44E+00/2.4E+00(+)
${f_{10}}$	2.92E−01/4.7E−01	**1.15E−01/4.9E−01(−)**	1.63E−01/7.2E−02(−)	1.88E+00/2.9E+00(+)	4.38E+01/5.5E+01(+)
${f_{11}}$	**1.08E+02/9.2E+01**	1.63E+02/1.1E+02(+)	1.50E+02/8.6E+01(+)	5.28E+02/1.1E+02(+)	1.65E+02/1.2E+02(+)
${f_{12}}$	**2.23E−01/6.2E−02**	2.31E−01/6.8E−02(+)	3.56E−01/8.0E−02(+)	4.50E−01/1.1E−01(+)	4.45E−01/2.0E−01(+)
${f_{13}}$	1.08E−01/2.1E−02	1.02E−01/2.3E−02(−)	1.71E−01/2.6E−02(+)	1.83E−01/3.1E−02(+)	**8.59E−02/3.1E−02(−)**
${f_{14}}$	**9.38E−02/2.4E−02**	9.49E−02/3.0E−02(+)	1.44E−01/2.6E−02(+)	1.86E−01/3.1E−02(+)	1.10E−01/6.1E−02(+)
${f_{15}}$	6.97E−01/1.4E−01	6.88E−01/1.7E−01(−)	1.00E+00/1.9E−01(+)	1.33E+00/2.2E−01(+)	**6.54E−01/2.4E−01(−)**
${f_{16}}$	**1.36E+00/2.9E−01**	1.66E+00/3.5E−01(+)	1.80E+00/3.2E−01(+)	2.72E+00/1.6E−01(+)	1.53E+00/5.2E−01(+)
${f_{17}}$	**8.35E−01/2.2E+00**	1.15E+05/1.3E+05(+)	1.82E+04/1.7E+04(+)	9.45E+03/6.1E+03(+)	1.36E+02/1.1E+02(+)
${f_{18}}$	**1.39E−01/1.6E−01**	5.88E+02/7.9E+02(+)	2.45E+03/1.4E+03(+)	3.67E+02/2.9E+02(+)	1.53E+01/1.8E+01(+)
${f_{19}}$	**8.14E−02/4.9E−02**	2.44E−01/1.5E−01(+)	3.95E−01/3.1E−01(+)	8.59E−01/2.4E−01(+)	8.83E−01/7.0E−01(+)
${f_{20}}$	**1.40E−01/1.7E−01**	4.12E+02/5.6E+02(+)	3.72E+02/3.6E+02(+)	1.14E+02/1.0E+02(+)	5.50E+00/6.9E+00(+)
${f_{21}}$	**3.17E−01/2.6E−01**	6.67E+03/7.3E+03(+)	2.82E+03/1.9E+03(+)	1.02E+03/9.4E+02(+)	8.34E+01/8.7E+01(+)
${f_{22}}$	**1.86E−01/1.8E−01**	5.06E−01/1.8E−01(+)	5.77E+00/1.8E+01(+)	5.96E−01/7.3E−01(+)	3.79E+01/4.8E+01(+)
${f_{23}}$	3.29E+02/2.9E−13	**2.82E+02/1.1E+02(−)**	3.17E+02/6.5E+01(−)	3.18E+02/4.8E+01(−)	3.29E+02/3.0E−13(=)
${f_{24}}$	**1.09E+02/2.6E+00**	1.13E+02/2.5E+00(+)	**1.09E+02/3.6E+00(=)**	1.16E+02/3.4E+00(+)	1.14E+02/4.7E+00(+)
${f_{25}}$	**1.23E+02/2.6E+01**	1.26E+02/5.6E+00(+)	1.34E+02/6.3E+00(+)	1.52E+02/7.3E+00(+)	1.82E+02/3.2E+01(+)
${f_{26}}$	1.00E+02/1.6E−02	1.00E+02/3.4E−02(=)	1.00E+02/2.4E−02(=)	1.00E+02/4.8E−02(=)	1.00E+02/3.3E−02(=)
${f_{27}}$	6.82E+01/1.5E+02	7.98E+01/1.4E+02(+)	4.69E+01/1.2E+02(−)	**3.38E+01/7.2E+01(−)**	2.43E+02/1.7E+02(+)
${f_{28}}$	3.76E+02/3.9E+01	3.64E+02/8.4E+00(−)	3.69E+02/8.9E+01(−)	**3.62E+02/4.9E+00(−)**	4.28E+02/6.0E+01(+)
${f_{29}}$	2.22E+02/5.2E−01	2.87E+02/3.5E+01(+)	4.04E+02/7.0E+01(+)	3.56E+02/5.4E+01(+)	**2.09E+02/5.8E+05(−)**
${f_{30}}$	**4.65E+02/1.1E+01**	5.17E+02/5.2E+01(+)	5.46E+02/4.6E+01(+)	5.23E+02/3.2E+01(+)	6.72E+02/2.0E+02(+)
**+/−/=**		21/2/7	20/3/7	23/2/5	22/4/4

**Note:**

The bold in the table represents the best result.

**Table 4:** Comparison with ABC variants under *D* = 30.

	ADDE	GABC	IABC	MABC	QABC
*f*	Mean/std	Mean/std	Mean/std	Mean/std	Mean/std
${f_1}$	**3.89E+04/3.7E+04**	8.13E+06/4.8E+06(+)	2.07E+07/3.9E+06(+)	6.65E+07/1.1E+07(+)	8.11E+04/6.3E+04(+)
${f_2}$	**2.23E−15/7.7E−15**	8.44E+01/1.7E+02(+)	7.21E+03/3.9E+03(+)	2.79E+01/4.3E+01(+)	3.46E−14/1.8E−14(+)
${f_3}$	**3.34E−15/1.4E−14**	5.09E+02/4.5E+02(+)	7.78E+02/3.2E+02(+)	4.69E+02/3.0E+02(+)	7.13E−14/3.0E−14(+)
${f_4}$	**1.60E+00/8.8E+00**	2.21E+01/2.8E+01(+)	7.86E+01/1.3E+01(+)	7.65E+01/1.5E+01(+)	1.04E+01/2.4E+01(+)
${f_5}$	**2.02E+01/4.3E−02**	2.03E+01/4.1E−02(+)	2.05E+01/4.9E−02(+)	2.09E+01/4.7E−02(+)	2.07E+01/4.6E−02(+)
${f_6}$	**1.94E+00/3.2E+00**	1.33E+01/1.4E+00(+)	9.34E+00/1.9E+00(+)	2.65E+01/1.1E+00(+)	6.29E+00/2.5E+00(+)
${f_7}$	1.16E−03/3.4E−03	9.40E−07/3.3E−06(−)	1.20E−04/2.1E−04(−)	**0.00E+00/0.0E+00(−)**	1.06E−02/1.4E−02(+)
${f_8}$	7.84E−12/5.5E−11	**0.00E+00/0.0E+00(−)**	8.31E−02/2.7E−01(+)	8.68E+01/8.0E+00(+)	1.00E+01/4.4E+00(+)
${f_9}$	**3.84E+01/9.9E+00**	4.79E+01/7.6E+00(+)	6.98E+01/9.2E+00(+)	1.84E+02/1.2E+01(+)	8.44E+01/3.1E+01(+)
${f_{10}}$	4.96E+00/3.1E+00	**6.46E−01/1.1E+00(−)**	2.45E+00/1.3E+00(−)	3.26E+03/1.8E+02(+)	2.01E+02/1.3E+02(+)
${f_{11}}$	1.84E+03/3.3E+02	**1.81E+03/3.2E+02(−)**	2.88E+03/3.7E+02(+)	6.15E+03/2.4E+02(+)	4.47E+03/5.4E+02(+)
${f_{12}}$	**2.81E−01/4.6E−02**	3.68E−01/6.8E−02(+)	6.95E−01/7.6E−02(+)	1.75E+00/1.5E−01(+)	1.13E+00/1.3E−01(+)
${f_{13}}$	2.69E−01/4.5E−02	**2.29E−01/3.0E−02(−)**	2.58E−01/3.0E−02(−)	4.26E−01/4.1E−02(+)	2.86E−01/5.6E−02(+)
${f_{14}}$	2.08E−01/2.9E−02	1.76E−01/2.1E−02(−)	**1.36E−01/1.8E−02(−)**	2.47E−01/2.9E−02(+)	3.02E−01/1.1E−01(+)
${f_{15}}$	**4.39E+00/8.0E−01**	5.49E+00/1.0E+00(+)	1.28E+01/1.4E+00(+)	1.75E+01/1.2E+00(+)	9.14E+00/2.2E+00(+)
${f_{16}}$	**9.28E+00/4.2E−01**	9.57E+00/4.3E−01(+)	1.05E+01/3.5E−01(+)	1.26E+01/1.9E−01(+)	1.08E+01/4.9E−01(+)
${f_{17}}$	**5.66E+02/1.8E+02**	2.10E+06/1.2E+06(+)	1.41E+06/5.6E+05(+)	1.69E+06/4.9E+05(+)	4.78E+03/4.4E+03(+)
${f_{18}}$	**1.36E+01/3.8E+00**	7.26E+03/6.4E+03(+)	1.90E+04/2.2E+04(+)	4.06E+02/1.4E+02(+)	1.98E+02/4.7E+02(+)
${f_{19}}$	**3.46E+00/6.7E−01**	6.64E+00/8.6E−01(+)	8.57E+00/1.5E+00(+)	9.25E+00/2.8E−01(+)	1.10E+01/1.6E+01(+)
${f_{20}}$	**1.02E+01/2.7E+00**	3.41E+03/1.9E+03(+)	2.70E+03/1.4E+03(+)	4.63E+03/1.5E+03(+)	7.51E+01/6.0E+01(+)
${f_{21}}$	**1.52E+02/1.0E+02**	3.69E+05/2.0E+05(+)	2.06E+05/7.0E+04(+)	2.22E+05/7.6E+04(+)	1.50E+03/2.0E+03(+)
${f_{22}}$	**4.03E+01/4.0E+01**	2.40E+02/1.0E+02(+)	2.27E+02/6.9E+01(+)	1.77E+02/5.1E+01(+)	2.59E+02/1.4E+02(+)
${f_{23}}$	3.15E+02/4.0E−13	3.15E+02/1.2E−01(=)	3.15E+02/3.6E−01(=)	3.15E+02/4.4E−13(=)	3.15E+02/4.5E−13(=)
${f_{24}}$	2.22E+02/4.5E+00	2.19E+02/1.7E+01(−)	**2.00E+02/3.1E−03(−)**	2.24E+02/1.5E+00(+)	2.34E+02/6.9E+00(+)
${f_{25}}$	**2.03E+02/1.3E−01**	2.07E+02/1.1E+00(+)	2.05E+02/0.0E+00(+)	2.15E+02/2.0E+00(+)	2.04E+02/1.6E+00(+)
${f_{26}}$	1.00E+02/4.1E−02	1.00E+02/4.6E−02(=)	1.00E+02/3.7E−02(=)	1.00E+02/5.2E−02(=)	1.12E+02/3.2E+01(+)
${f_{27}}$	**3.67E+02/4.8E+01**	4.00E+02/5.6E+01(+)	4.05E+02/1.7E+00(+)	5.00E+02/2.1E+01(+)	5.10E+02/9.2E+01(+)
${f_{28}}$	**8.02E+02/2.6E+01**	8.32E+02/4.7E+01(+)	8.93E+02/4.7E+01(+)	9.44E+02/2.0E+01(+)	1.10E+03/2.8E+02(+)
${f_{29}}$	**7.17E+02/8.1E+00**	1.26E+03/2.8E+02(+)	3.56E+03/1.7E+03(+)	2.37E+03/6.4E+02(+)	1.11E+06/3.1E+06(+)
${f_{30}}$	**7.27E+02/2.7E+02**	2.83E+03/8.8E+02(+)	5.40E+03/9.4E+02(+)	4.52E+03/6.2E+02(+)	1.97E+03/1.0E+03(+)
**+/−/=**		21/2/7	23/2/5	27/2/1	29//1/0

**Note:**

The bold in the table represents the best result.

**Table 5:** Comparison with ABC variants under *D* = 50.

	ADDE	GABC	IABC	MABC	QABC
*f*	Mean/std	Mean/std	Mean/std	Mean/std	Mean/std
${f_1}$	8.86E+05/3.7E+05	1.11E+07/3.9E+06(+)	1.84E+07/3.4E+06(+)	2.55E+08/2.9E+07(+)	**1.81E+05/1.2E+05(−)**
${f_2}$	8.14E+02/2.4E+03	6.99E+03/6.9E+03(+)	1.93E+04/1.4E+04(+)	3.78E+03/3.5E+03(+)	**1.18E−09/2.9E−09(−)**
${f_3}$	2.52E−02/1.7E−01	5.63E+03/1.8E+03(+)	2.87E+03/8.4E+02(+)	9.86E+04/8.6E+03(+)	**8.37E−07/2.6E−06(−)**
${f_4}$	7.21E+01/3.0E+01	5.67E+01/3.0E+01(−)	1.33E+02/3.3E+01(+)	9.64E+01/2.1E+00(+)	**5.21E+01/3.8E+01(−)**
${f_5}$	**2.03E+01/4.0E−02**	2.05E+01/4.6E−02(+)	2.06E+01/4.6E−02(+)	2.11E+01/3.6E−02(+)	2.08E+01/4.3E−02(+)
${f_6}$	**1.43E+01/1.0E+01**	2.78E+01/2.3E+00(+)	1.94E+01/2.4E+00(+)	5.74E+01/1.3E+00(+)	1.93E+01/3.6E+00(+)
${f_7}$	1.45E−04/1.0E−03	3.99E−04/1.3E−03(+)	8.38E−03/1.2E−02(+)	**0.00E+00/0.0E+00(−)**	8.82E−03/1.4E−02(+)
${f_8}$	3.80E−11/1.9E−10	**0.00E+00/0.0E+00(−)**	1.44E+00/2.7E+00(+)	2.56E+02/1.2E+01(+)	1.86E+01/6.8E+00(+)
${f_9}$	**8.91E+01/1.3E+01**	1.16E+02/1.5E+01(+)	1.63E+02/1.5E+01(+)	4.21E+02/1.4E+01(+)	1.89E+02/4.9E+01(+)
${f_{10}}$	8.64E+00/3.7E+00	**1.99E+00/1.2E+00(−)**	6.20E+00/1.9E+00(−)	8.74E+03/3.3E+02(+)	3.78E+02/2.5E+02(+)
${f_{11}}$	4.44E+03/6.0E+02	**4.39E+03/5.7E+02(−)**	6.60E+03/6.2E+02(+)	1.26E+04/3.6E+02(+)	8.60E+03/5.9E+02(+)
${f_{12}}$	**2.83E−01/5.5E−02**	4.64E−01/6.6E−02(+)	8.41E−01/1.2E−01(+)	2.85E+00/2.2E−01(+)	1.21E+00/1.4E−01(+)
${f_{13}}$	3.83E−01/4.1E−02	**2.73E−01/2.7E−02(−)**	4.43E−01/3.2E−02(+)	5.86E−01/3.6E−02(+)	3.73E−01/7.7E−02(−)
${f_{14}}$	2.78E−01/1.1E−01	**2.13E−01/2.3E−02(−)**	2.18E−01/1.7E−02(−)	3.00E−01/2.7E−02(+)	4.24E−01/2.1E−01(+)
${f_{15}}$	**1.01E+01/1.4E+00**	1.45E+01/2.3E+00(+)	3.09E+01/2.4E+00(+)	4.08E+01/1.7E+00(+)	2.58E+01/5.4E+00(+)
${f_{16}}$	**1.76E+01/4.7E−01**	1.83E+01/5.3E−01(+)	1.91E+01/4.4E−01(+)	2.24E+01/1.8E−01(+)	1.98E+01/4.2E−01(+)
${f_{17}}$	1.70E+04/1.5E+04	5.84E+06/2.3E+06(+)	5.19E+06/1.1E+06(+)	1.35E+07/1.7E+06(+)	**1.19E+04/8.2E+03(−)**
${f_{18}}$	**4.90E+01/1.7E+01**	4.71E+03/3.4E+03(+)	4.43E+04/8.5E+04(+)	1.37E+03/8.9E+02(+)	6.34E+02/1.0E+03(+)
${f_{19}}$	**1.06E+01/8.0E−01**	1.58E+01/1.8E+00(+)	1.49E+01/6.3E+00(+)	3.19E+01/7.5E+00(+)	1.35E+01/9.7E+00(+)
${f_{20}}$	**4.34E+01/3.4E+01**	1.51E+04/4.0E+03(+)	6.29E+03/2.2E+03(+)	4.84E+04/8.5E+03(+)	1.96E+02/9.6E+01(+)
${f_{21}}$	**1.78E+03/1.7E+03**	2.61E+06/1.2E+06(+)	1.56E+06/4.6E+05(+)	4.20E+06/1.1E+06(+)	3.20E+03/1.7E+03(+)
${f_{22}}$	**3.54E+02/1.9E+02**	7.91E+02/1.9E+02(+)	7.93E+02/1.4E+02(+)	1.00E+03/1.3E+02(+)	6.75E+02/3.0E+02(+)
${f_{23}}$	3.44E+02/4.5E−13	3.44E+02/5.2E−01(=)	3.44E+02/4.9E−13(=)	3.44E+02/4.6E−13(=)	3.44E+02/4.8E−13(=)
${f_{24}}$	2.69E+02/3.7E+00	2.57E+02/5.7E−01(−)	**2.00E+02/2.0E−03(−)**	2.58E+02/1.1E+00(−)	2.80E+02/3.6E+00(+)
${f_{25}}$	2.06E+02/6.4E−01	2.15E+02/1.5E+00(+)	**2.00E+02/0.0E+00(−)**	2.53E+02/5.0E+00(+)	2.10E+02/4.7E+00(+)
${f_{26}}$	1.08E+02/2.7E+01	**1.00E+02/4.0E−02(−)**	1.49E+02/5.0E+01(+)	1.01E+02/4.6E−02(−)	1.10E+02/5.0E+01(+)
${f_{27}}$	**4.17E+02/1.0E+02**	8.82E+02/3.1E+02(+)	7.53E+02/9.6E+01(+)	1.57E+03/7.7E+01(+)	9.06E+02/1.1E+02(+)
${f_{28}}$	**1.19E+03/8.4E+01**	1.26E+03/4.6E+01(+)	1.46E+03/1.1E+02(+)	1.49E+03/3.7E+01(+)	2.13E+03/6.1E+02(+)
${f_{29}}$	**8.89E+02/1.4E+02**	1.79E+03/3.6E+02(+)	3.44E+03/9.0E+02(+)	3.22E+04/1.5E+04(+)	4.95E+06/1.4E+07(+)
${f_{30}}$	**8.51E+03/4.6E+02**	9.57E+03/7.5E+02(+)	1.26E+04/1.3E+03(+)	1.63E+04/1.7E+03(+)	1.15E+04/2.6E+03(+)
**+/−/=**		21/1/8	25/1/4	26/1/3	23/1/6

**Note:**

The bold in the table represents the best result.

According to the statistical results, compared with GABC, ADDE algorithm has similar improvement results in different dimensions, with 21 benchmarks achieving better results. Compared with QABC, ADDE shows the best improvement results in 30D, among which 29 benchmarks are achieving better results and one benchmark is similar, while none of the 30 benchmarks becomes worse Although it is more difficult to deal with high-dimensional problems, compare with IABC, ADDE is superior in 20, 23 and 25 benchmarks of 10D, 30D and 50D respectively. The higher the benchmark of dimension, the better its performance is Finally, the improvement results of ADDE at 10D and 50D were similar to those of MABC, obtaining 26 better benchmarks, and the improvement results were slightly better at 30D, obtaining 27 better benchmarks. In general, the performance of our algorithm outperforms all the comparative ABC variants, especially when dealing with some more complex high-dimensional problems.

### Comparison between ADDE and DE variants

In this section, we compare with the advanced DE variants include DE/rand/1, RBDE, DEGL, SADE, QUATRE, S-QUATRE and QUATRE-DEG. The experimental results are recorded in Tables 6–11. As shown in Tables 6–11, first of all, in terms of the 10D real number optimization, the novel ADDE performs better than DE/rand/1 and RBDE on 25 benchmark functions and performs similar on two benchmark functions, better than SADE in 21 benchmark functions and similar in six benchmark functions, better than QUATRE-DEG in 26 functions and similar in two functions, and better or similar than DEGL, QUATRE, and S-QUATRE on all 30 benchmark functions. Then, in terms of the 30D real number optimization, the novel ADDE performs better than DE/RAND/1 in 27 benchmark functions and is similar in two benchmark functions, better than SADE in 26 functions and similar in one function, better than QUATRE-DEG in 24 functions and similar in three functions, and better or similar than RBDE, DEGL, QUATRE, and S-QUATRE in all 30 benchmark functions. Finally, in terms of the 50D real number optimization, our algorithm performs better than DE/rand/1, RBDE, and DEGL on 28 benchmark functions and performs similar on one function, better than SADE in 24 functions and similar in two functions, better than QUATRE-DEG in 23 functions and similar in one function, better than QUATRE on 23 benchmark functions and performs similar on one function, better than S-QUATRE on 23 benchmark functions and performs similarly on two functions. Therefore, compared with these DE variants, we still get the best performance.

**Table 6:** Comparison with DE variants under *D* = 10.

	ADDE	DE/rand/1	RBDE	DEGL	SADE
*f*	Mean/std	Mean/std	Mean/std	Mean/std	Mean/std
${f_1}$	**1.62E−13/2.0E−13**	9.42E+04/2.7E+04(+)	1.53E+05/5.5E+04(+)	6.06E+02/1.2E+03(+)	5.02E−15/2.3E−14(−)
${f_2}$	**0.00E+00/0.0E+00**	4.29E−01/1.7E−01(+)	2.75E+01/1.0E+01(+)	1.07E−10/6.3E−10(+)	**0.00E+00/0.0E+00(=)**
${f_3}$	**0.00E+00/0.0E+00**	1.00E−03/4.5E−04(+)	5.70E−02/2.1E−02(+)	1.62E−10/8.0E−10(+)	**0.00E+00/0.0E+00(=)**
${f_4}$	1.53E+01/1.7E+01	1.49E+01/1.6E+01(−)	**9.94E+00/1.4E+01(−)**	2.68E+01/1.5E+01(+)	2.13E+01/1.7E+01(+)
${f_5}$	**1.61E+01/6.7E+00**	2.02E+01/8.4E−02(+)	2.02E+01/2.4E−01(+)	1.86E+01/4.5E+00(+)	1.90E+01/4.0E+00(+)
${f_6}$	**0.00E+00/0.0E+00**	4.27E−02/5.9E−02(+)	8.91E−01/3.7E−01(+)	5.26E−02/1.7E−01(+)	**0.00E+00/0.0E+00(=)**
${f_7}$	**1.47E−02/2.1E−02**	3.47E−01/6.8E−02(+)	3.79E−01/5.9E−02(+)	3.86E−02/3.6E−02(+)	1.90E−02/2.6E−02(+)
${f_8}$	**0.00E+00/0.0E+00**	7.02E+00/1.3E+00(+)	8.61E+00/1.4E+00(+)	2.64E+00/1.2E+00(+)	**0.00E+00/0.0E+00(=)**
${f_9}$	**3.63E+00/1.1E+00**	2.14E+01/3.1E+00(+)	2.19E+01/3.2E+00(+)	5.61E+00/1.8E+00(+)	1.09E+01/2.1E+00(+)
${f_{10}}$	**2.92E−01/4.7E−01**	8.42E+01/2.3E+01(+)	1.15E+02/2.6E+01(+)	8.60E+01/6.2E+01(+)	2.78E+01/1.0E+01(+)
${f_{11}}$	**1.08E+02/9.2E+01**	8.57E+02/1.5E+02(+)	8.75E+02/1.8E+02(+)	3.57E+02/1.4E+02(+)	6.27E+02/1.5E+02(+)
${f_{12}}$	**2.32E−01/6.2E−02**	7.09E−01/1.2E−01(+)	7.15E−01/1.3E−01(+)	4.41E−01/9.7E−02(+)	6.76E−01/1.3E−01(+)
${f_{13}}$	**1.08E−01/2.1E−02**	2.09E−01/3.8E−02(+)	2.27E−01/3.8E−02(+)	1.20E−01/2.4E−02(+)	1.45E−01/2.6E−02(+)
${f_{14}}$	**9.38E−02/2.4E−02**	1.77E−01/2.7E−02(+)	1.70E−01/3.1E−02(+)	1.59E−01/4.8E−02(+)	1.59E−01/3.9E−02(+)
${f_{15}}$	**6.97E−01/1.4E−01**	2.18E+00/3.2E−01(+)	2.28E+00/3.2E−01(+)	7.62E−01/1.5E−01(+)	1.40E+00/2.3E−01(+)
${f_{16}}$	**1.36E+00/2.9E−01**	2.83E+00/2.1E−01(+)	2.89E+00/1.6E−01(+)	2.13E+00/3.1E−01(+)	2.38E+00/2.6E−01(+)
${f_{17}}$	**8.35E−01/2.2E+00**	1.65E+02/4.2E+01(+)	2.54E+02/7.4E+01(+)	9.41E+01/6.2E+01(+)	2.66E+01/2.4E+01(+)
${f_{18}}$	**1.39E−01/1.6E−01**	6.05E+00/1.2E+00(+)	7.59E+00/1.5E+00(+)	2.46E+00/1.0E+00(+)	7.03E−01/5.0E−01(+)
${f_{19}}$	**8.14E−02/4.9E−02**	8.21E−01/1.8E−01(+)	1.05E+00/2.0E−01(+)	8.34E−01/5.5E−01(+)	4.15E−01/1.3E−01(+)
${f_{20}}$	**1.40E−01/1.7E−01**	1.97E+00/4.3E−01(+)	2.56E+00/4.9E−01(+)	9.35E−01/4.1E−01(+)	3.97E−01/1.9E−01(+)
${f_{21}}$	**3.17E−01/2.6E−01**	7.75E+00/2.6E+00(+)	1.27E+01/5.0E+00(+)	1.85E+00/2.7E+00(+)	7.04E−01/3.5E−01(+)
${f_{22}}$	**1.86E−01/1.8E−01**	2.25E+00/1.8E+00(+)	9.45E+00/2.2E+00(+)	8.57E+00/8.6E+00(+)	2.68E+00/1.4E+00(+)
${f_{23}}$	3.29E+02/2.9E−13	3.29E+02/4.1E−13(=)	3.29E+02/1.6E−09(=)	3.29E+02/2.9E−13(=)	3.29E+02/2.9E−13(=)
${f_{24}}$	**1.09E+02/2.6E+00**	1.30E+02/3.9E+00(+)	1.30E+02/4.0E+00(+)	**1.09E+02/3.8E+00(=)**	1.14E+02/3.0E+00(+)
${f_{25}}$	1.23E+02/2.6E+01	1.70E+02/1.5E+01(+)	1.80E+02/1.2E+01(+)	1.27E+02/9.9E+00(+)	**1.22E+02/1.3E+01(−)**
${f_{26}}$	1.00E+02/1.6E−02	1.00E+02/3.5E−02(=)	1.00E+02/4.2E−02(=)	1.00E+02/1.9E−02(=)	1.00E+02/3.3E−02(=)
${f_{27}}$	6.82E+01/1.5E+02	2.08E+01/7.1E+01(−)	**1.03E+01/4.3E+01(−)**	9.07E+01/1.6E+02(+)	5.69E+01/1.3E+02(−)
${f_{28}}$	3.76E+02/3.9E+01	**3.67E+02/7.1E+00(−)**	3.70E+02/6.3E+00(−)	4.02E+02/5.5E+01(+)	3.78E+02/2.4E+01(+)
${f_{29}}$	**2.22E+02/5.2E−01**	3.83E+02/3.4E+01(+)	4.27E+02/4.4E+01(+)	2.31E+02/6.6E+00(+)	2.23E+02/8.0E+00(+)
${f_{30}}$	**4.65E+02/1.1E+01**	5.63E+02/3.0E+01(+)	6.18E+02/3.4E+01(+)	5.92E+02/8.1E+01(+)	4.80E+02/2.4E+01(+)
**+/−/=**		25/2/3	25/2/3	27/3/0	21/6/3

**Note:**

The bold in the table represents the best result.

**Table 7:** Comparison with DE variants under *D* = 30.

	ADDE	DE/rand/1	RBDE	DEGL	SADE
*f*	Mean/std	Mean/std	Mean/std	Mean/std	Mean/std
${f_1}$	**3.89E+04/3.7E+04**	8.89E+07/1.9E+07(+)	1.03E+08/2.1E+07(+)	5.08E+05/6.7E+05(+)	1.04E+05/8.2E+04(+)
${f_2}$	**2.23E−15/7.7E−15**	1.76E+03/5.2E+02(+)	8.12E+04/1.7E+04(+)	2.84E−14/0.0E+00(+)	2.45E−14/9.9E−15(+)
${f_3}$	**3.34E−15/1.4E−14**	2.59E+01/6.3E+00(+)	1.62E+02/2.8E+01(+)	3.10E−09/1.4E−08(+)	**5.46E−14/1.1E−14(+)**
${f_4}$	**1.60E+00/8.8E+00**	1.22E+02/6.7E+00(+)	1.51E+02/7.6E+00(+)	3.25E+01/3.6E+01(+)	7.33E+00/2.0E+01(+)
${f_5}$	**2.02E+01/4.3E−02**	2.09E+01/4.8E−02(+)	2.09E+01/5.1E−02(+)	2.08E+01/7.8E−02(+)	2.09E+01/5.7E−02(+)
${f_6}$	1.94E+00/3.2E+00	3.03E+01/1.1E+00(+)	3.13E+01/1.2E+00(+)	6.02E+00/1.9E+00(+)	**6.60E−01/8.6E−01(−)**
${f_7}$	1.16E−03/3.4E−03	3.83E−02/7.8E−02(+)	3.93E−01/1.4E−01(+)	1.23E−02/1.4E−02(+)	**6.27E−04/3.6E−03(−)**
${f_8}$	**7.84E−12/5.5E−11**	1.20E+02/7.2E+00(+)	1.21E+02/8.3E+00(+)	4.68E+01/1.1E+01(+)	1.37E−01/3.5E−01(+)
${f_9}$	**3.84E+01/9.9E+00**	1.96E+02/1.0E+01(+)	1.97E+02/1.1E+01(+)	6.86E+01/1.6E+01(+)	5.69E+01/2.9E+01(+)
${f_{10}}$	**4.96E+00/3.1E+00**	3.86E+03/2.4E+02(+)	3.99E+03/1.9E+02(+)	2.42E+03/4.3E+02(+)	3.25E+02/5.0E+01(+)
${f_{11}}$	**1.84E+03/3.3E+02**	6.58E+03/2.2E+02(+)	6.52E+03/3.0E+02(+)	3.84E+03/5.1E+02(+)	5.74E+03/2.6E+02(+)
${f_{12}}$	**2.81E−01/4.6E−02**	2.03E+00/1.9E−01(+)	2.08E+00/2.4E−01(+)	1.22E+00/2.2E−01(+)	1.92E+00/2.1E−01(+)
${f_{13}}$	**2.69E−01/4.5E−02**	4.79E−01/5.4E−02(+)	4.98E−01/5.4E−02(+)	2.82E−01/5.1E−02(+)	2.73E−01/3.7E−02(+)
${f_{14}}$	**2.08E−01/2.9E−02**	2.92E−01/3.5E−02(+)	3.00E−01/4.7E−02(+)	2.33E−01/3.5E−02(+)	2.47E−01/3.5E−02(+)
${f_{15}}$	**4.39E+00/8.0E−01**	1.90E+01/1.2E+00(+)	1.97E+01/1.1E+00(+)	1.04E+01/2.8E+00(+)	1.01E+01/1.5E+00(+)
${f_{16}}$	**9.28E+00/4.2E−01**	1.25E+01/2.1E−01(+)	1.25E+01/2.1E−01(+)	1.14E+01/2.9E−01(+)	1.20E+01/2.5E−01(+)
${f_{17}}$	**5.66E+02/1.8E+02**	2.41E+06/7.0E+05(+)	2.67E+06/8.2E+05(+)	8.70E+04/6.1E+04(+)	1.68E+03/4.9E+02(+)
${f_{18}}$	**1.36E+01/3.8E+00**	2.92E+04/1.7E+04(+)	6.84E+04/3.0E+04(+)	3.19E+02/1.8E+02(+)	7.48E+01/2.4E+01(+)
${f_{19}}$	**3.46E+00/6.7E−01**	1.03E+01/6.1E−01(+)	1.14E+01/5.7E−01(+)	1.25E+01/1.2E+01(+)	4.79E+00/6.7E−01(+)
${f_{20}}$	**1.02E+01/2.7E+00**	4.61E+02/8.3E+01(+)	8.62E+02/2.0E+02(+)	9.97E+01/2.9E+01(+)	3.49E+01/2.0E+01(+)
${f_{21}}$	**1.52E+02/1.0E+02**	2.02E+05/6.9E+04(+)	3.33E+05/8.7E+04(+)	7.55E+03/5.8E+03(+)	4.24E+02/2.2E+02(+)
${f_{22}}$	**4.03E+01/4.0E+01**	2.17E+02/6.3E+01(+)	2.32E+02/7.3E+01(+)	2.08E+02/6.4E+01(+)	1.15E+02/6.3E+01(+)
${f_{23}}$	3.15E+02/4.0E−13	3.15E+02/8.4E−05(=)	3.15E+02/2.2E−03(=)	3.15E+02/4.5E−13(=)	3.15E+02/4.0E−13(=)
${f_{24}}$	2.22E+02/4.5E+00	**2.08E+02/2.5E+00(−)**	2.24E+02/2.8E+00(+)	2.28E+02/4.8E+00(+)	2.25E+02/2.5E+00(+)
${f_{25}}$	**2.03E+02/1.3E−01**	2.23E+02/2.7E+00(+)	2.25E+02/3.2E+00(+)	2.10E+02/3.2E+00(+)	2.05E+02/3.3E+00(+)
${f_{26}}$	1.00E+02/4.1E−02	1.00E+02/5.1E−02(=)	1.00E+02/5.6E−02(=)	1.26E+02/4.4E+01(+)	1.02E+02/1.4E+01(+)
${f_{27}}$	3.67E+02/4.8E+01	3.90E+02/3.5E+01(+)	6.80E+02/7.0E+01(+)	4.54E+02/7.4E+01(+)	**3.58E+02/4.6E+01(−)**
${f_{28}}$	**8.02E+02/2.6E+01**	9.75E+02/2.3E+01(+)	1.00E+03/2.2E+01(+)	9.38E+02/1.2E+02(+)	8.32E+02/3.0E+01(+)
${f_{29}}$	**7.17E+02/8.1E+00**	1.12E+04/2.7E+03(+)	1.78E+04/4.1E+03(+)	1.34E+03/4.5E+02(+)	8.47E+02/9.0E+01(+)
${f_{30}}$	**7.27E+02/2.7E+02**	5.70E+03/8.8E+02(+)	7.55E+03/1.1E+03(+)	2.13E+03/7.0E+02(+)	9.82E+02/3.2E+02(+)
**+/−/=**		27/2/1	28/2/0	29/1/0	26/1/3

**Note:**

The bold in the table represents the best result.

**Table 8:** Comparison with DE variants under *D* = 50.

	ADDE	DE/rand/1	RBDE	DEGL	SADE
*f*	Mean/std	Mean/std	Mean/std	Mean/std	Mean/std
${f_1}$	8.86E+05/3.7E+05	3.41E+08/3.6E+07(+)	3.75E+08/5.7E+07(+)	1.11E+06/5.3E+05(+)	**5.28E+05/2.7E+05(−)**
${f_2}$	**8.14E+02/2.4E+03**	6.79E+07/3.0E+07(+)	2.75E+08/7.1E+07(+)	8.54E+02/1.4E+03(+)	3.63E+03/2.6E+03(+)
${f_3}$	**2.52E−02/1.7E−01**	6.92E+04/6.6E+03(+)	7.96E+04/7.6E+03(+)	2.19E+03/2.0E+03(+)	6.77E−02/1.3E−01(+)
${f_4}$	7.21E+01/3.0E+01	1.26E+02/6.7E+00(+)	2.04E+02/1.6E+01(+)	7.05E+01/4.2E+01(−)	**6.35E+01/3.9E+01(−)**
${f_5}$	**2.03E+01/4.0E−02**	2.11E+01/3.1E−02(+)	2.11E+01/3.5E−02(+)	2.10E+01/5.0E−02(+)	2.11E+01/3.4E−02(+)
${f_6}$	1.43E+01/1.0E+01	5.89E+01/1.7E+00(+)	6.04E+01/1.6E+00(+)	2.05E+01/3.4E+00(+)	**7.35E+00/2.4E+00(−)**
${f_7}$	**1.45E−04/1.0E−03**	7.42E−01/4.3E−02(+)	1.02E+00/1.4E−02(+)	1.49E−02/3.6E−02(+)	5.56E−03/5.7E−03(+)
${f_8}$	**3.80E−11/1.9E−10**	2.83E+02/8.9E+00(+)	2.86E+02/9.6E+00(+)	9.65E+01/2.7E+01(+)	2.73E+00/1.6E+00(+)
${f_9}$	8.91E+01/1.3E+01	4.10E+02/1.4E+01(+)	4.23E+02/1.7E+01(+)	1.44E+02/2.2E+01(+)	**5.64E+01/1.1E+01(−)**
${f_{10}}$	**8.64E+00/3.7E+00**	9.16E+03/4.2E+02(+)	9.26E+03/3.1E+02(+)	5.89E+03/8.1E+02(+)	6.61E+02/1.1E+02(+)
${f_{11}}$	**4.44E+03/6.0E+02**	1.30E+04/3.4E+02(+)	1.29E+04/3.0E+02(+)	8.19E+03/8.4E+02(+)	1.12E+04/4.6E+02(+)
${f_{12}}$	**2.83E−01/5.5E−02**	3.19E+00/2.5E−01(+)	3.12E+00/3.2E−01(+)	1.87E+00/2.8E−01(+)	2.76E+00/2.4E−01(+)
${f_{13}}$	**3.83E−01/4.1E−02**	6.76E−01/4.1E−02(+)	6.94E−01/5.6E−02(+)	4.91E−01/7.1E−02(+)	3.90E−01/4.7E−02(+)
${f_{14}}$	**2.78E−01/1.1E−01**	3.78E−01/1.1E−01(+)	4.05E−01/8.0E−02(+)	3.12E−01/3.6E−02(+)	3.10E−01/2.7E−02(+)
${f_{15}}$	**1.01E+01/1.4E+00**	4.61E+01/2.4E+00(+)	6.43E+01/5.9E+00(+)	3.47E+01/7.9E+00(+)	1.64E+01/6.9E+00(+)
${f_{16}}$	**1.76E+01/4.7E−01**	2.22E+01/2.4E−01(+)	2.23E+01/2.2E−01(+)	2.08E+01/4.5E−01(+)	2.17E+01/2.2E−01(+)
${f_{17}}$	**1.70E+04/1.5E+04**	1.66E+07/4.2E+06(+)	1.91E+07/4.3E+06(+)	5.78E+05/5.0E+05(+)	1.89E+04/1.3E+04(+)
${f_{18}}$	**4.90E+01/1.7E+01**	5.64E+04/2.6E+04(+)	1.43E+05/5.8E+04(+)	9.12E+02/7.7E+02(+)	3.81E+02/2.6E+02(+)
${f_{19}}$	**1.06E+01/8.0E−01**	2.66E+01/1.9E+00(+)	3.30E+01/3.8E+00(+)	3.09E+01/2.6E+01(+)	2.46E+01/1.5E+01(+)
${f_{20}}$	**4.34E+01/3.4E+01**	2.15E+04/4.0E+03(+)	2.62E+04/6.0E+03(+)	5.81E+02/2.2E+02(+)	2.97E+02/9.2E+01(+)
${f_{21}}$	**1.78E+03/1.7E+03**	7.13E+06/2.0E+06(+)	8.01E+06/1.9E+06(+)	2.71E+05/1.2E+05(+)	6.27E+03/4.1E+03(+)
${f_{22}}$	**3.54E+02/1.9E+02**	1.24E+03/1.4E+02(+)	1.28E+03/1.5E+02(+)	6.91E+02/1.7E+02(+)	5.33E+02/1.5E+02(+)
${f_{23}}$	3.44E+02/4.5E−13	3.44E+02/5.4E−04(=)	3.44E+02/4.0E−03(=)	3.44E+02/5.0E−13(=)	3.44E+02/4.6E−13(=)
${f_{24}}$	**2.69E+02/3.7E+00**	2.70E+02/2.3E+00(+)	2.86E+02/1.8E+00(+)	2.79E+02/4.4E+00(+)	2.72E+02/1.9E+00(+)
${f_{25}}$	**2.06E+02/6.4E−01**	2.73E+02/8.3E+00(+)	2.79E+02/8.2E+00(+)	2.21E+02/7.6E+00(+)	2.08E+02/9.1E+00(+)
${f_{26}}$	1.08E+02/2.7E+01	**1.01E+02/5.5E−02(−)**	**1.01E+02/5.8E−02(−)**	1.84E+02/3.7E+01(+)	1.49E+02/5.0E+01(+)
${f_{27}}$	**4.17E+02/1.0E+02**	1.50E+03/6.0E+01(+)	1.63E+03/4.0E+01(+)	9.06E+02/9.4E+01(+)	5.03E+02/5.7E+01(+)
${f_{28}}$	**1.19E+03/8.4E+01**	1.51E+03/3.6E+01(+)	1.54E+03/6.6E+01(+)	1.73E+03/3.7E+02(+)	**1.19E+03/7.4E+01(=)**
${f_{29}}$	**8.89E+02/1.4E+02**	1.31E+05/3.1E+04(+)	2.10E+05/6.4E+04(+)	9.39E+05/6.7E+06(+)	1.23E+03/1.8E+02(+)
${f_{30}}$	**8.51E+03/4.6E+02**	3.18E+04/6.8E+03(+)	4.95E+04/1.1E+04(+)	1.18E+04/1.1E+03(+)	9.97E+03/8.7E+02(+)
**+/−/=**		28/1/1	28/1/1	28/1/1	24/2/4

**Note:**

The bold in the table represents the best result.

**Table 9:** Comparison with DE variants under *D* = 10.

	ADDE	QUATRE	S-QUATRE	QUATRE-DEG
*f*	Mean/std	Mean/std	Mean/std	Mean/std
${f_1}$	**1.62E−13/2.0E−13**	6.59E−08/7.5E−08(+)	1.86E−02/9.5E−03(+)	2.84E−01/2.0E−01(+)
${f_2}$	**0.00E+00/0.0E+00**	**0.00E+00/0.0E+00(=)**	8.71E−12/6.3E−12(+)	5.82E+00/2.9E+01(+)
${f_3}$	**0.00E+00/0.0E+00**	**0.00E+00/0.0E+00(=)**	4.46E−15/1.5E−14(+)	1.14E−03/3.8E−04(+)
${f_4}$	**1.53E+01/1.7E+01**	2.17E+01/1.6E+01(+)	1.57E+01/1.7E+01(+)	2.36E+01/1.6E+01(+)
${f_5}$	**1.61E+01/6.7E+00**	1.90E+01/4.8E+00(+)	2.01E+01/1.9E−01(+)	1.96E+01/2.8E+00(+)
${f_6}$	**0.00E+00/0.0E+00**	4.09E−01/6.2E−01(+)	2.06E−01/5.7E−01(+)	2.33E−01/3.6E−01**(+)**
${f_7}$	**1.47E−02/2.1E−02**	1.03E−01/6.1E−02(+)	2.46E−01/6.7E−02(+)	9.98E−02/4.8E−02(+)
${f_8}$	**0.00E+00/0.0E+00**	3.06E+00/1.7E+00(+)	6.26E+00/9.5E−01(+)	2.69E+00/1.5E+00(+)
${f_9}$	**3.63E+00/1.1E+00**	9.35E+00/4.4E+00(+)	1.81E+01/2.8E+00(+)	7.92E+00/4.1E+00(+)
${f_{10}}$	**2.92E−01/4.7E−01**	6.07E+01/6.3E+01(+)	1.61E+02/5.9E+01(+)	4.23E+01/6.4E+01(+)
${f_{11}}$	**1.08E+02/9.2E+01**	3.32E+02/2.2E+02(+)	8.87E+02/1.3E+02(+)	3.48E+02/2.1E+02(+)
${f_{12}}$	2.32E−01/6.2E−02	2.75E−01/1.9E−01(+)	7.52E−01/1.2E−01(+)	**2.30E−02/2.6E−02**(−)
${f_{13}}$	**1.08E−01/2.1E−02**	1.48E−01/6.1E−02(+)	1.54E−01/3.2E−02(+)	1.17E−01/3.2E−02(+)
${f_{14}}$	**9.38E−02/2.4E−02**	1.58E−01/5.2E−02(+)	1.26E−01/3.6E−02(+)	1.07E−01/4.2E−02(+)
${f_{15}}$	**6.97E−01/1.4E−01**	1.06E+00/4.2E−01(+)	1.69E+00/2.9E−01(+)	8.24E−01/2.7E−01(+)
${f_{16}}$	**1.36E+00/2.9E−01**	1.94E+00/5.5E−01(+)	2.71E+00/2.1E−01(+)	1.79E+00/5.6E−01(+)
${f_{17}}$	8.35E−01/2.2E+00	9.87E+01/6.7E+01(+)	9.78E+01/6.1E+01(+)	**8.07E+01/7.4E+01**(−)
${f_{18}}$	**1.39E−01/1.6E−01**	3.87E+00/4.4E+00(+)	3.57E+00/8.9E−01(+)	2.74E+00/2.9E+00(+)
${f_{19}}$	**8.14E−02/4.9E−02**	7.46E−01/7.6E−01(+)	8.48E−01/4.8E−01(+)	3.94E−01/4.6E−01(+)
${f_{20}}$	**1.40E−01/1.7E−01**	1.92E+00/2.5E+00(+)	1.97E+00/3.6E−01(+)	1.09E+00/8.2E−01(+)
${f_{21}}$	**3.17E−01/2.6E−01**	5.19E+01/6.2E+01(+)	1.05E+01/2.5E+01(+)	3.13E+01/4.7E+01(+)
${f_{22}}$	**1.86E−01/1.8E−01**	1.60E+01/3.0E+01(+)	1.30E+01/7.6E+00(+)	1.15E+01/1.1E+01(+)
${f_{23}}$	3.29E+02/2.9E−13	3.29E+02/2.9E−13(=)	3.29E+02/2.9E−13(=)	3.29E+02/8.1E−07(=)
${f_{24}}$	**1.09E+02/2.6E+00**	1.19E+02/5.8E+00(+)	1.26E+02/3.0E+00(+)	1.19E+02/4.9E+00(+)
${f_{25}}$	**1.23E+02/2.6E+01**	1.76E+02/3.7E+01(+)	1.58E+02/3.3E+01(+)	1.73E+02/3.8E+01(+)
${f_{26}}$	1.00E+02/1.6E−02	1.00E+02/7.0E−02(=)	1.00E+02/2.9E−02(=)	1.00E+02/4.1E−02(=)
${f_{27}}$	**6.82E+01/1.5E+02**	2.30E+02/1.7E+02(+)	8.35E+01/1.6E+02(+)	2.25E+02/1.8E+02(+)
${f_{28}}$	**3.76E+02/3.9E+01**	4.32E+02/6.0E+01(+)	4.15E+02/5.7E+01(+)	4.06E+02/5.4E+01(+)
${f_{29}}$	**2.22E+02/5.2E−01**	1.22E+05/4.9E+05(+)	7.06E+02/3.5E+03(+)	4.20E+04/3.0E+05(+)
${f_{30}}$	**4.65E+02/1.1E+01**	5.46E+02/1.3E+02(+)	5.18E+02/1.3E+02(+)	5.18E+02/7.5E+01(+)
**+/−/=**		26/4/0	28/2/0	26/2/2

**Note:**

The bold in the table represents the best result.

**Table 10:** Comparison with DE variants under *D* = 30.

	ADDE	QUATRE	S-QUATRE	QUATRE-DEG
***f***	**Mean/std**	**Mean/std**	**Mean/std**	**Mean/std**
${f_1}$	**3.89E+04/3.7E+04**	1.37E+05/1.0E+05(+)	1.22E+05/9.4E+04(+)	1.15E+05/8.1E+04(+)
${f_2}$	**2.23E−15/7.7E−15**	1.39E−14/1.4E−14(+)	2.23E−14/1.2E−14(+)	1.86E+01/5.1E+01(+)
${f_3}$	**3.34E−15/1.4E−14**	3.34E−14/2.8E−14(+)	6.91E−14/2.4E−14(+)	3.84E−03/5.8E−04(+)
${f_4}$	**1.60E+00/8.8E+00**	2.53E+00/1.2E+01(+)	4.86E+00/1.6E+01(+)	3.78E+00/1.5E+01(+)
${f_5}$	2.02E+01/4.3E−02	2.05E+01/9.4E−02(+)	2.07E+01/4.9E−02(+)	**2.00E+01/4.1E−02**(−)
${f_6}$	**1.94E+00/3.2E+00**	3.69E+00/2.3E+00(+)	6.64E+00/9.3E+00(+)	2.60E+00/2.0E+00(+)
${f_7}$	**1.16E−03/3.4E−03**	7.77E−03/1.2E−02(+)	2.47E−03/3.8E−03(+)	5.90E−03/6.3E−03(+)
${f_8}$	**7.84E−12/5.5E−11**	2.85E+01/7.1E+00(+)	5.99E+01/2.8E+00(+)	2.20E+01/5.4E+00(+)
${f_9}$	**3.84E+01/9.9E+00**	7.07E+01/2.0E+01(+)	1.50E+02/6.2E+00(+)	5.53E+01/1.4E+01(+)
${f_{10}}$	**4.96E+00/3.1E+00**	4.99E+02/2.5E+02(+)	2.25E+03/1.7E+02(+)	3.86E+02/1.8E+02(+)
${f_{11}}$	**1.84E+03/3.3E+02**	3.35E+03/6.9E+02(+)	5.51E+03/2.1E+02(+)	2.41E+03/6.5E+02(+)
${f_{12}}$	2.81E−01/4.6E−02	4.43E−01/2.3E−01(+)	1.43E+00/1.5E−01(+)	**8.04E−02/3.7E−02**(−)
${f_{13}}$	**2.69E−01/4.5E−02**	3.10E−01/8.4E−02(+)	3.60E−01/4.2E−02(+)	2.82E−01/7.2E−02(+)
${f_{14}}$	**2.08E−01/2.9E−02**	3.38E−01/1.3E−01(+)	2.37E−01/2.9E−02(+)	2.80E−01/1.1E−01(+)
${f_{15}}$	4.39E+00/8.0E−01	7.41E+00/2.1E+00(+)	1.37E+01/6.8E−01(+)	**3.97E+00/1.0E+00**(−)
${f_{16}}$	**9.28E+00/4.2E−01**	1.03E+01/6.8E−01(+)	1.21E+01/2.5E−01(+)	1.03E+01/6.3E−01(+)
${f_{17}}$	**5.66E+02/1.8E+02**	2.31E+03/2.6E+03(+)	1.49E+03/4.8E+02(+)	1.68E+03/8.1E+02(+)
${f_{18}}$	**1.36E+01/3.8E+00**	8.77E+01/4.6E+01(+)	1.02E+02/3.7E+01(+)	6.05E+01/3.4E+01(+)
${f_{19}}$	**3.46E+00/6.7E−01**	4.75E+00/1.4E+00(+)	5.79E+00/7.8E−01(+)	4.11E+00/1.5E+00(+)
${f_{20}}$	**1.02E+01/2.7E+00**	3.89E+01/3.7E+01(+)	4.49E+01/5.8E+00(+)	2.57E+01/1.6E+01(+)
${f_{21}}$	**1.52E+02/1.0E+02**	4.94E+02/2.7E+02(+)	9.94E+02/2.0E+02(+)	4.60E+02/2.0E+02(+)
${f_{22}}$	**4.03E+01/4.0E+01**	3.14E+02/1.5E+02(+)	3.05E+02/1.0E+02(+)	2.91E+02/1.9E+02(+)
${f_{23}}$	3.15E+02/4.0E−13	3.15E+02/4.0E−13(=)	3.15E+02/4.0E−13(=)	3.15E+02/4.4E−07(=)
${f_{24}}$	**2.22E+02/4.5E+00**	2.28E+02/5.8E+00(+)	2.23E+02/6.4E+00(+)	2.24E+02/3.9E+00(+)
${f_{25}}$	2.03E+02/1.3E−01	2.03E+02/8.4E−01(=)	2.03E+02/3.0E−01(=)	2.03E+02/4.7E−01(=)
${f_{26}}$	1.00E+02/4.1E−02	1.00E+02/1.0E−01(=)	1.00E+02/4.2E−02(=)	1.00E+02/2.0E+01(=)
${f_{27}}$	**3.67E+02/4.8E+01**	4.17E+02/7.1E+01(+)	3.84E+02/6.3E+01(+)	3.86E+02/5.3E+01(+)
${f_{28}}$	**8.02E+02/2.6E+01**	9.20E+02/1.5E+02(+)	9.37E+02/8.1E+01(+)	8.51E+02/7.6E+01(+)
${f_{29}}$	**7.17E+02/8.1E+00**	1.81E+05/1.3E+06(+)	7.28E+02/2.4E+01(+)	1.70E+05/1.2E+06(+)
${f_{30}}$	**7.27E+02/2.7E+02**	1.80E+03/7.5E+02(+)	1.30E+03/5.3E+02(+)	1.53E+03/7.5E+02(+)
**+/−/=**		27/3/0	27/3/0	24/3/3

**Note:**

The bold in the table represents the best result.

**Table 11:** Comparison with DE variants under *D* = 50.

	ADDE	QUATRE	S-QUATRE	QUATRE-DEG
*f*	Mean/std	Mean/std	Mean/std	Mean/std
${f_1}$	8.86E+05/3.7E+05	**6.64E+05/3.1E+05(−)**	1.37E+06/5.0E+05(+)	8.15E+05/3.3E+05(+)
${f_2}$	8.14E+02/2.4E+03	**1.46E−04/3.4E−04(−)**	2.41E−01/3.1E−01(−)	6.95E+03/6.5E+03(+)
${f_3}$	2.52E−02/1.7E−01	**3.16E−04/9.1E−04(−)**	5.91E−03/5.4E−03(−)	2.73E−01/2.0E−01(+)
${f_4}$	7.21E+01/3.0E+01	5.05E+01/4.2E+01(−)	7.14E+01/3.2E+01(−)	**4.96E+01/4.1E+01**(−)
${f_5}$	2.03E+01/4.0E−02	2.07E+01/7.6E−02(+)	2.09E+01/2.3E−02(+)	**2.00E+01/9.3E−02**(−)
${f_6}$	1.43E+01/1.0E+01	1.13E+01/4.3E+00(−)	**8.30E+00/1.4E+01(−)**	8.63E+00/4.6E+00(−)
${f_7}$	**1.45E−04/1.0E−03**	2.27E−03/5.2E−03(+)	1.88E−03/4.4E−03(+)	6.60E−03/5.5E−03(+)
${f_8}$	**3.80E−11/1.9E−10**	7.09E+01/1.4E+01(+)	1.54E+02/5.4E+00(+)	5.91E+01/1.4E+01(+)
${f_9}$	**8.91E+01/1.3E+01**	1.59E+02/3.9E+01(+)	3.25E+02/1.1E+01(+)	1.12E+02/2.3E+01(+)
${f_{10}}$	**8.64E+00/3.7E+00**	1.69E+03/5.2E+02(+)	5.86E+03/2.3E+02(+)	1.36E+03/3.5E+02(+)
${f_{11}}$	**4.44E+03/6.0E+02**	7.14E+03/1.0E+03(+)	1.12E+04/2.3E+02(+)	5.07E+03/8.8E+02(+)
${f_{12}}$	2.83E−01/5.5E−02	4.72E−01/2.5E−01(+)	1.99E+00/1.3E−01(+)	**1.62E−01/6.2E−02**(−)
${f_{13}}$	**3.83E−01/4.1E−02**	4.64E−01/1.0E−01(+)	4.71E−01/5.9E−02(+)	3.94E−01/5.7E−02(+)
${f_{14}}$	**2.78E−01/1.1E−01**	4.39E−01/2.1E−01(+)	3.54E−01/1.9E−01(+)	4.34E−01/2.2E−01(+)
${f_{15}}$	1.01E+01/1.4E+00	1.69E+01/4.3E+00(+)	2.85E+01/1.3E+00(+)	**8.42E+00/2.2E+00**(−)
${f_{16}}$	**1.76E+01/4.7E−01**	1.99E+01/8.2E−01(+)	2.17E+01/1.7E−01(+)	1.94E+01/9.8E−01(+)
${f_{17}}$	1.70E+04/1.5E+04	2.75E+04/1.4E+04(+)	**7.96E+03/4.4E+03(−)**	2.35E+04/1.4E+04(+)
${f_{18}}$	**4.90E+01/1.7E+01**	2.42E+02/1.9E+02(+)	3.09E+02/6.9E+01(+)	8.88E+02/9.2E+02(+)
${f_{19}}$	1.06E+01/8.0E−01	**8.88E+00/2.4E+00(−)**	1.43E+01/3.4E+00(+)	8.65E+00/2.0E+00(−)
${f_{20}}$	**4.34E+01/3.4E+01**	1.32E+02/6.8E+01(+)	1.37E+02/2.3E+01(+)	1.03E+02/6.3E+01(+)
${f_{21}}$	**1.78E+03/1.7E+03**	9.09E+03/7.4E+03(+)	2.58E+03/1.2E+03(+)	5.63E+03/4.5E+03(+)
${f_{22}}$	**3.54E+02/1.9E+02**	1.07E+03/2.9E+02(+)	1.11E+03/1.8E+02(+)	8.76E+02/2.4E+02(+)
${f_{23}}$	3.44E+02/4.5E−13	3.44E+02/4.4E−13(=)	3.44E+02/4.3E−13(=)	3.44E+02/2.2E−07(=)
${f_{24}}$	**2.69E+02/3.7E+00**	2.76E+02/3.5E+00(+)	2.72E+02/2.7E+00(+)	2.74E+02/2.9E+00(+)
${f_{25}}$	**2.06E+02/6.4E−01**	2.07E+02/1.7E+00(+)	**2.06E+02/8.6E−01(=)**	2.07E+02/1.4E+00(+)
${f_{26}}$	1.08E+02/2.7E+01	1.28E+02/6.5E+01(+)	1.16E+02/3.7E+01(+)	1.25E+02/5.4E+01(+)
${f_{27}}$	**4.17E+02/1.0E+02**	6.58E+02/9.0E+01(+)	4.51E+02/5.9E+01(+)	5.69E+02/8.9E+01(+)
${f_{28}}$	**1.19E+03/8.4E+01**	1.44E+03/2.7E+02(+)	1.36E+03/2.0E+02(+)	1.33E+03/2.8E+02(+)
${f_{29}}$	**8.89E+02/1.4E+02**	1.97E+06/9.9E+06(+)	9.36E+02/1.3E+02(+)	7.21E+05/5.1E+06(+)
${f_{30}}$	**8.51E+03/4.6E+02**	9.94E+03/8.8E+02(+)	9.13E+03/6.9E+02(+)	9.54E+03/9.8E+02(+)
**+/−/=**		23/1/6	23/2/5	23/1/6

**Note:**

The bold in the table represents the best result.

According to the statistical results, at 10D and 30D, ADDE improved extremely well compared with DEGL, QUATRE and S-QUATRE, and none of the 30 benchmarks got worse. However, the effect was not so good in dealing with higher dimension (50D) benchmarks, and some benchmarks still got worse. In addition, both ADDE and RBDE improved better than DE/RAND/1 and RBDE on high-dimensional problems, both achieving 28 better and a similar result on the 50D basis. Secondly, compared with the improved results of SADE, which is also an adaptive scheme, ADDE has the best effect on the basis of 30D, achieving 26 better results and one similar results. It only becomes worse on F6, F7 and F27. Finally, the improvement of ADDE over advanced QUATRE-DEG deteriorates as the dimension increase, becoming best at 10D, obtaining 26 better benchmarks, and 24 and 23 better benchmarks at 30D and 50D, respectively. It can be analyzed that compared with the more advanced DE variants, the effect on the high-dimensional benchmark is slightly decreased. This is because ADDE algorithm has enhanced certain search ability (from ABC), so the development energy will be relatively weaker, and some high-dimensional problems need to be developed more. Overall, however, we still got the best performance compared to these DE variants.

### Comparison of convergence

**Figure 1:**
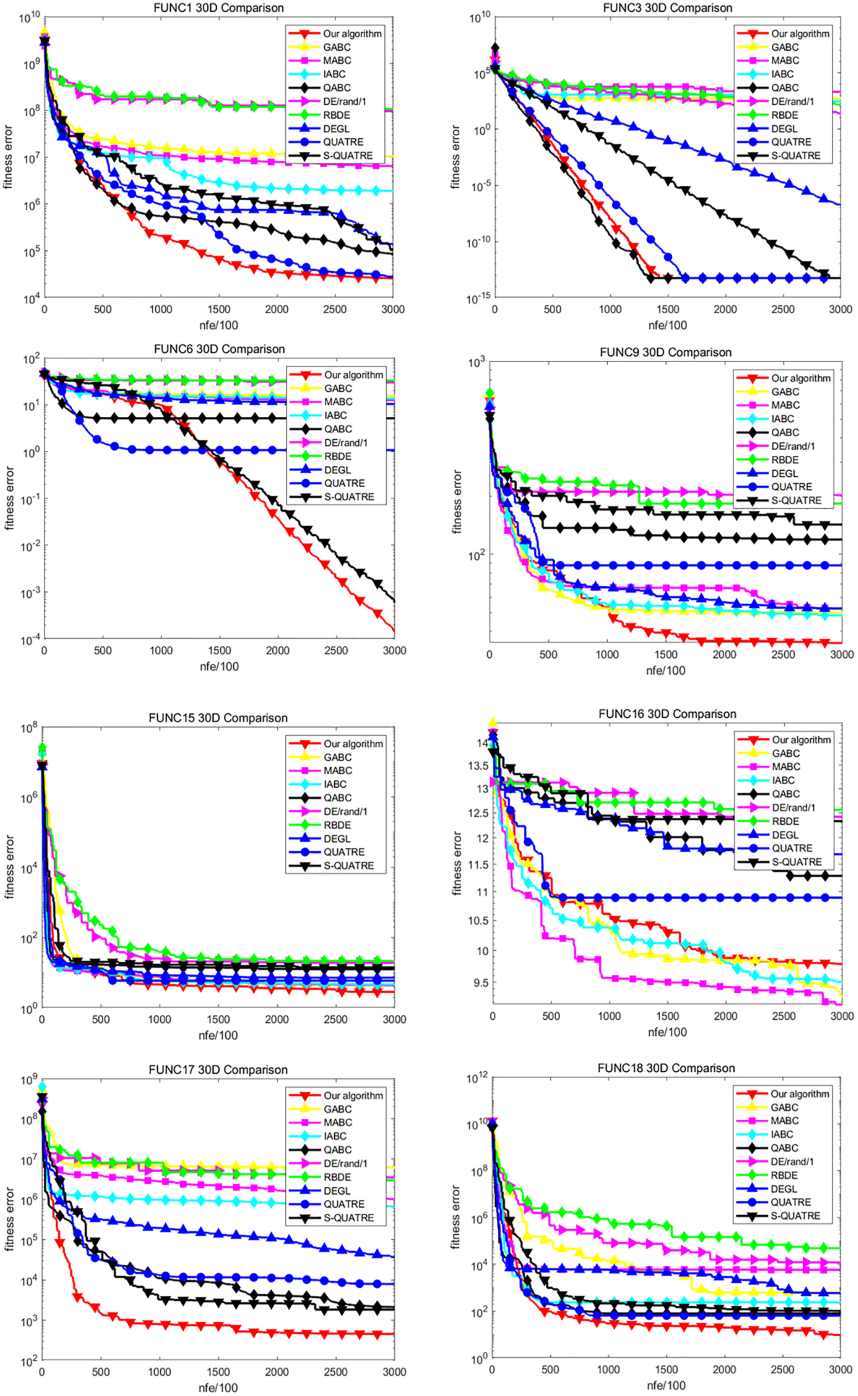
Convergence comparison on some functions with *D* = 30 on CEC2014.

**Figure 2:**
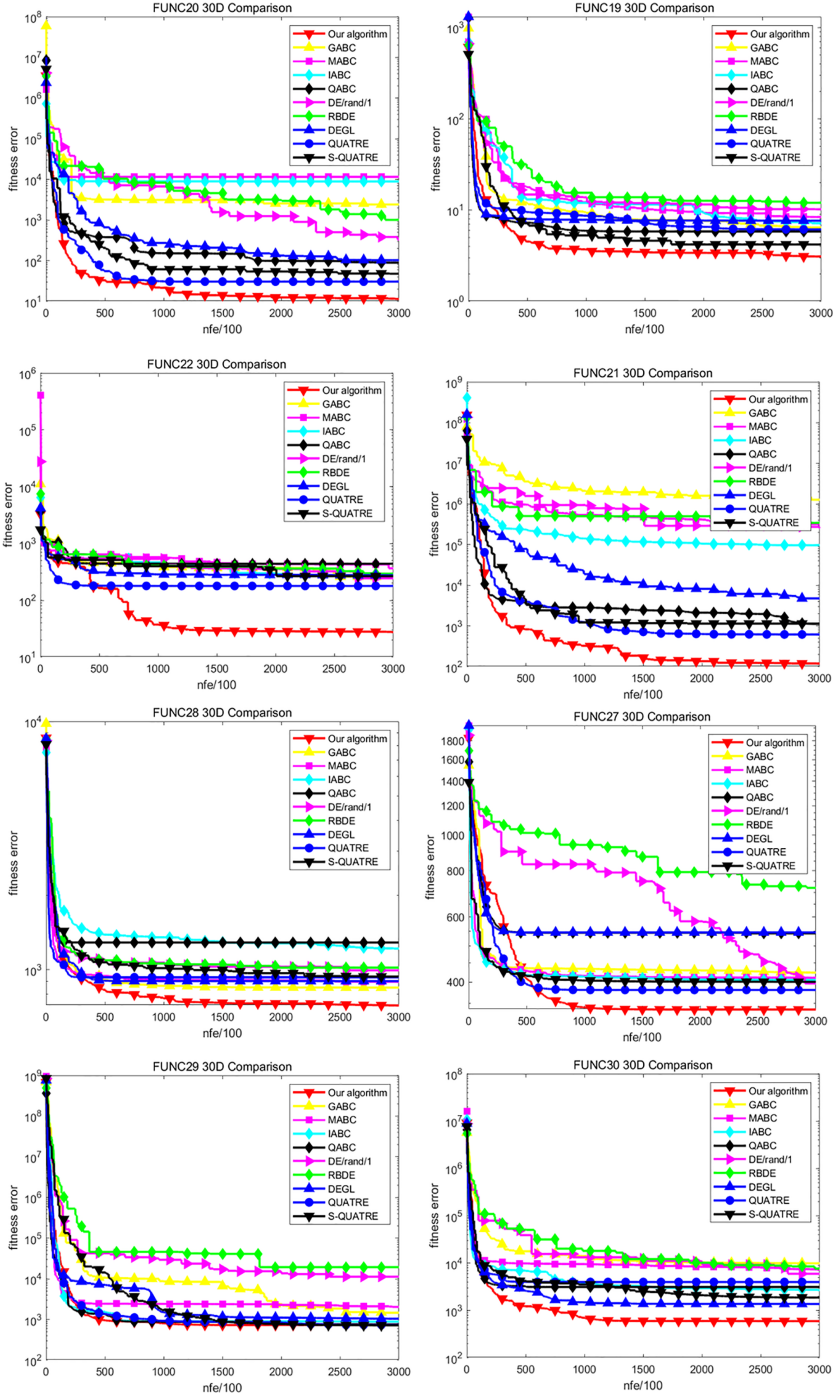
Convergence comparison on some functions with *D* = 30 on CEC2014.

We select the representatives of four benchmark functions in the CEC2014 test suite and analyze their convergence rate, including basic single-mode function FUNC1, FUNC3, basic multi-mode function FUNC6, FUNC9, FUNC15, FUNC16, mixed function FUNC17–FUNC22, combined function FUNC27–FUNC30. Figures 1 and 2 are convergence curves of intermediate values of all the compared algorithms running 51 times independently under 30D. As shown in the figure, the convergence speed of DE variant is better in FUNC1 and FUNC3 as a whole. It can be noted that our algorithm converges fastest and reaches the global optimal solution. In FUNC6, except for our ADDE and S-QUATRE, other algorithms converge prematurely on this function and fall into the local optimal solution. Our ADDE algorithm and S-QUATRE perform particularly well on this function because they are still searching for a better solution quickly. More importantly, our ADDE performs better than S-QUATRE at the current number of iterations. On the basis of FUNC9 and FUNC15, the convergence speed of the new ADDE is slightly better than that of other algorithms. At present, they have converged, and our ADDE has achieved higher accuracy. On the basis of FUNC16, the ADDE is slightly worse than several ABC variants, but it still performs better than DE variants. In the comparison of the convergence speed of all mixed functions from FUNC17 to FUNC22, our algorithm is stronger than all other algorithms and has the fastest convergence speed. This is because the solution of mixed function needs not only search but also development. It can be seen that our ADDE algorithm achieves a good balance between development and search. The performance of the ADDE on the combinatorial function is still bright. The convergence performance of FUNC27, FUNC28 and FUNC30 is better than that of all other algorithms. On the benchmark of FUNC29, it converges to a similar global optimum with QABC, which is slightly better than S-QUATRE, QUATRE and IABC, while GABC, MABC, DE and RBDE converge too early and fall into a local optimum. In general, our algorithm ADDE has achieved the best results in comparison, so it is competitive in terms of convergence speed.

### Comparison of complexity

We evaluate the algorithm complexity of the novel ADDE on 30D, and the algorithm complexity of the CEC2014 is given in Liang, Qu & Suganthan (2014). The running time of the given arithmetic expression is denoted as ${T_0}$. The time spent in evaluating the f18 function in the CEC214 test set for 200,000 times is recorded as ${T_1}$. The optimization algorithm performs 200,000 evaluations on the f18 function completely, and the time consumed is recorded as ${T_2}$. We evaluated ${T_2}$ 5 times and got the average time $\widehat {{T_2}}$. Accordingly, the complexity $(\widehat {{T_2}} - {T_1})/{T_0}$ can be obtained. The comparison results are recorded in Table 12. As shown in Table 9, compared with some DE variants, the novel ADDE will take more time, the sorting behavior of ADDE and the new one-dimensional update equation increase the complexity of the algorithm. but we know that the improvement of the algorithm will inevitably add some code, which is tolerable. Compared with all ABC variants and DEGL, ADDE has better time complexity.

**Table 12:** Complexity comparison on 30-D optimization.

Algorithms	${T_0}$	${T_1}$	$\widehat {{T_2}}$	$\displaystyle{{\widehat {{T_2}} - {T_1}} \over {{T_0}}}$
GABC	0.0924	0.3590	2.7015	25.3517
MABC	0.0924	0.3590	5.3996	54.5519
IABC	0.0924	0.3590	4.3876	43.5996
QABC	0.0924	0.3590	5.9012	59.9805
DE/rand/1	0.0924	0.3590	1.4027	11.2954
RBDE	0.0924	0.3590	1.4298	11.5887
DEGL	0.0924	0.3590	4.2051	41.6245
QUATRE	0.0924	0.3590	1.7701	15.2716
S-QUATRE	0.0924	0.3590	1.9237	16.9340
ADDE	0.0924	0.3590	2.4777	22.9296

## Conclusion

DE is a simple and powerful algorithm, which has attracted more and more attention in recent years. We know that the outstanding development ability of DE is largely due to its multidimensional search strategy, which enables it to quickly approach the global optimal solution. But for a better solution, the one-dimensional search strategy can ensure a greater update success rate. Especially in the late stage of the optimization iteration, the one-dimensional search strategy can slow down the particles falling into the local optimal solution. Based on this, this article proposes an adaptive updating scheme, which uses multi-dimensional and one-dimensional updating strategies to achieve a better balance between search and development. The multi-dimensional updating strategy comes from the improvement of the standard DE algorithm, while the one-dimensional updating strategy comes from the improvement of the standard ABC algorithm. The main idea to improve them is to increase elitism which is used to improve the overall optimization speed. To verify the performance of our ADDE algorithm, we compared it with other famous ABC variants and DE variants on the CEC2014 dataset. The results show that the new algorithm is competitive. In the future, we will further use ADDE to solve practical engineering problems (Sung, Sun & Chang, 2020; Sung, Zhao & Chang, 2020; Xu et al., 2020; Wang et al., 2020).

## Supplemental Information

10.7717/peerj-cs.1007/supp-1 Supplemental Information 1 Code.

## Additional Information and Declarations

### Data Availability

The following information was supplied regarding data availability: The code of our proposed algorithm compared with other algorithms are available in the Supplemental Files.
